# Gilded vaterite optothermal transport in a bubble

**DOI:** 10.1038/s41598-023-39068-8

**Published:** 2023-07-27

**Authors:** Hod Gilad, Hani Barhum, Andrey Ushkov, Andrey Machnev, Daniel Ofer, Vjačeslavs Bobrovs, Pavel Ginzburg

**Affiliations:** 1 Department of Electrical Engineering, Tel Aviv University, 69978 Ramat Aviv, Tel Aviv, Israel; 2 Light-Matter Interaction Centre, Tel Aviv University, 69978 Tel Aviv, Israel; 3 Triangle Regional Research and Development Center, 3007500 Kfar Qara, Israel; 4 Institute of Telecommunications, Riga Technical University, Azenes Street 12, Riga, 1048 Latvia

**Keywords:** Optical physics, Nanoscale materials

## Abstract

Laser beams, capable of controlling the mechanical motion of micron-scale objects, can serve as a tool, enabling investigations of numerous interaction scenarios under full control. Beyond pure electromagnetic interactions, giving rise to conventional gradient forces and radiation pressure, environment-induced thermal effects can play a role and, in certain cases, govern the dynamics. Here we explore a thermocapillary Marangoni effect, which is responsible for creating long-range few hundreds of nano-Newton forces, acting on a bubble around a ‘gilded vaterite’ nanoparticle. Decorating calcium carbonate spherulite (the vaterite) with gold nanoseeds allows tuning its optical absorption and, as a result, controlling its temperature in a solution. We demonstrate that keeping a balance between electromagnetic and thermal interactions allows creating of a stable micron-scale bubble around the particle and maintaining its size over time. The bubbles are shown to remain stable over minutes even after the light source is switched off. The bubbles were shown to swim toward a laser focus for over 400-µm distances across the sample. Optothermal effects, allowing for efficient transport, stable bubble creation, and particle–fluid interaction control, can grant nano-engineered drug delivery capsules with additional functions toward a theragnostic paradigm shift.

## Introduction

Multifunctional nanoparticles find use in numerous applications, including precision medicine^1^, where miniature theragnostic devices are foreseen to provide targeted and efficient treatment. One, among a large set of requirements for a successful performance, is particle stability inside an environment, where undesired capsule breakdown or dissolution can significantly affect therapeutic action. A variety of different techniques to control particle–fluid interactions were demonstrated with polymer cap layer protection being among the widely used ones, e.g.^2^. However, in the case of drug–cell interactions on a single entity level, different concepts are also in place, as they allow for fast and easy prototyping under full control. Optical tweezers are a convenient tool for controlling interaction on a micron-scale level, matching typical dimensions of cell and mesoscopic capsules for drug delivery. Since the first demonstration^3^, optomechanical manipulation became a widely used tool in microbiological and biomedical studies^4–6^ even going down to the molecular level^7^. Appreciating the length scales diversity of potential optomechanical applications, it is worth noting the laser cooling of atoms^8^ as one extreme and visionary solar sails of macroscopic satellites, e.g.^9^ as another.

**Figure 1:**
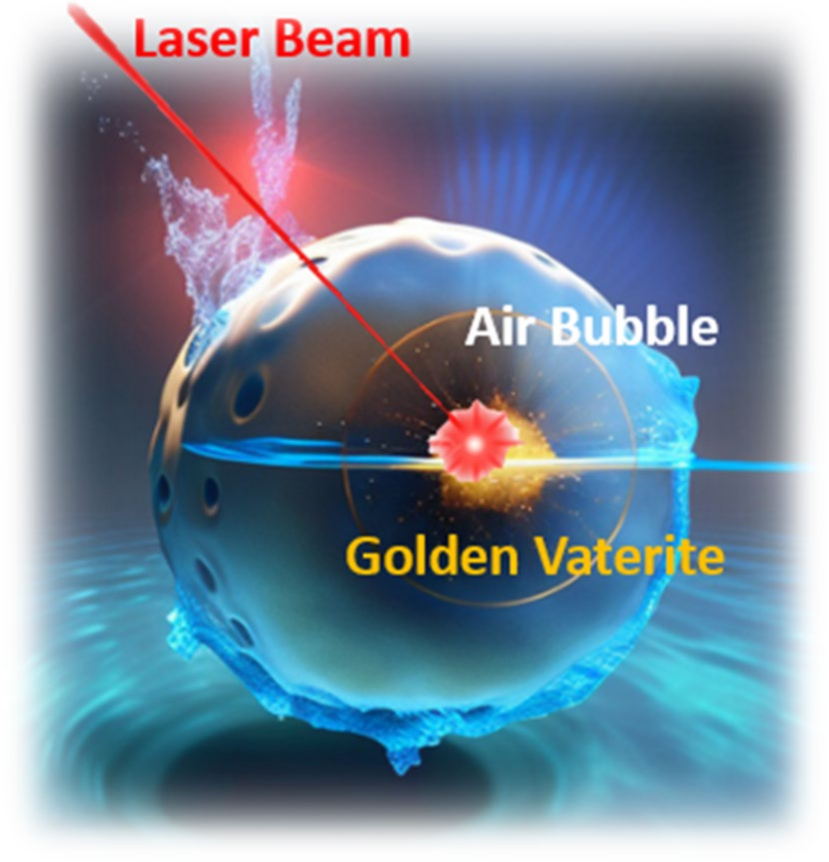
Schematic representation of a vaterite optomechanical transport in a bubble—the laser beam heats a resonant structure and creates an air bubble around it, which then becomes subject to optomechanical and optothermal manipulation.

Here we will demonstrate an efficient laser-assisted manipulation of nanoengineered micron-scale particles, capable of encapsulating functional materials for drug delivery applications. For controlling particle–fluid interaction, the optomechanical transport will be organized in an air bubble, which prevents the cargo dissolution, thus virtually controlling the drug realize rate. To realize this new scenario, several essential properties have to be engineered, including a biocompatible particle design, which allows effective bubble inflation and thermo-optical forces to control particle transport. For this application, inorganic polycrystals are favorable, as accommodating both biochemical and optical properties made possible within the same platform. Among a range of inorganic particles^10,11^, calcium carbonate (CaCO_3_) has numerous unambiguous advantages owing to its availability, low-cost facile fabrication, environmental safety, biocompatibility, and biodegradability^12^. CaCO_3_ has three crystalline forms—vaterite, calcite, and aragonite ^13^. Vaterite undergoes a phase transition upon an interaction with a solvent and can dissociate or become calcite, e.g.^14^. In both of those scenarios, functional materials, stored in the pores, will be released. While this mechanism is the key to realizing drug delivery scenarios, spontaneous uncontrollable cargo release is highly undesirable. Apart from polymer encapsulation^15^, vaterite phase transformation can be inhibited by preventing its direct contact with the fluid via a bubble. However, pure vaterite is a low-contrast transparent material with an anisotropic refractive index of ~ 1.5 to 1.7^16^, which cannot be efficiently heated without the excessive use of high-power laser beams. Recall, that the refractive index constant has to be assessed versus an embedding medium, i.e., an aqueous solution. Apart from technical challenges, high-power beams might pose limitations in biological studies, causing cells’ phototoxicity or photodamage^17^. As a result, introducing contrast and absorbing materials into the vaterite cargo can provide a solution. Decorating vaterite with plasmonic nanoparticles allows tuning low-refractive index cargo into a functional biogenic metamaterial—golden vaterite ^18^. Depending on the fabrication protocol, gold nanoresonators can either be infused into the volume of the cargo or decorate its surface, thus controlling the optical properties of the compound mesoscopic particle. However, if an insufficiently small number of gold nanoparticles are attached to/loaded into the vaterite, the laser heating will be inefficient. On the other hand, excessive doping with gold will increase the radiation pressure, pushing the particle from the optical trap. Balancing between these two extremes will be shown to provide a stable bubble formation and will allow for its transport. Figure 1 demonstrates the concept of the golden vaterite optomechanical transport in a bubble.

The manuscript is organized as follows: the fabrication of gilded vaterite nanoparticles will be discussed first, and then followed by an investigation of particles with the aid of dark-field spectroscopy to retrieve their optical properties. Bubble formation will be studied next and then followed by their optothermal manipulation, which is demonstrated before the Conclusion.

## Particle synthesis and gold decoration-gilded vaterite

**Figure 2:**
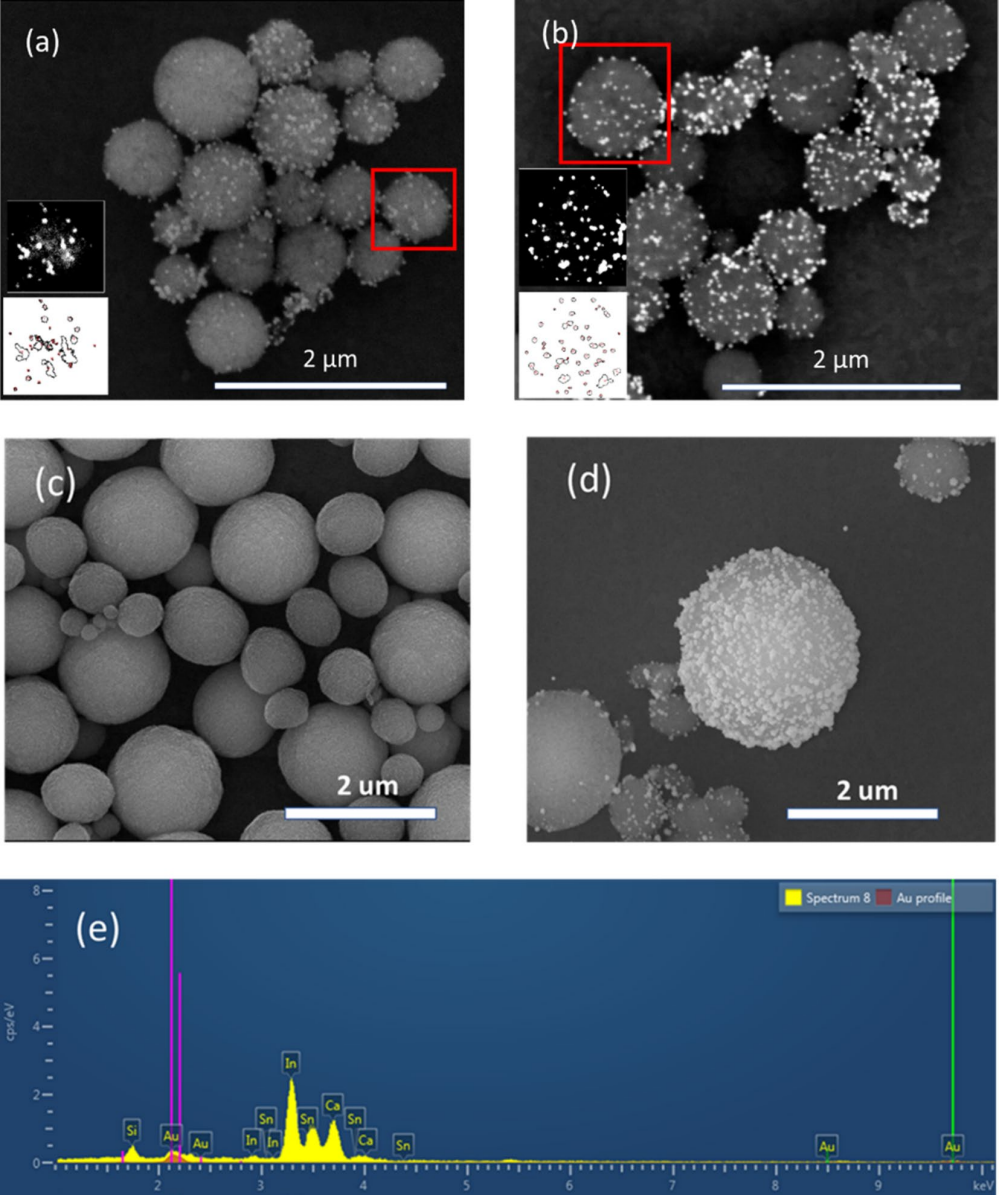
(**a**) SEM image of vaterite particles, coated with gold nanoseeds. The bottom-left corner insets are: the top—the contrast image of the particle of interest, and the bottom—the gold nanoparticles identified and measured by image processing software. (**b**) Vaterite-coated particles with higher coverage of gold nanoparticles, including insets of the contrast image and the software-identified particles. (**c**) The initial vaterite particles before the gold coverage. (**d**) The particles after gold addition, illustrating the changes in the morphology. (**e**) Energy-dispersive X-ray spectroscopy (EDS) spectrum, confirming the presence of gold in the bright particles.

Vaterite particles were synthesized following a previously established method, e.g.^19,20^. In brief, the synthesis involves preparing a solution consisting of 85% ethylene glycol (EG) and 15% water, with a 1:5 ratio of CaCl_2_ to Na_2_CO_3_ and a 0.015 M concentration of CaCl_2_. This mixture facilitated the formation of vaterite particles with the desired characteristics. To attach gold nanoseeds to the surface of the synthesized vaterite particles, a polyvinylpyrrolidone (PVP) layer was first introduced. This was achieved by adding 2 mg of vaterite to 2 mL of an aqueous solution containing PVP. The mixture was then vortexed for 2 h to ensure thorough coverage of the vaterite particles with the PVP layer. Following this step, the particles were washed using centrifugation to remove any excess PVP. Subsequently, 3 mL of ethanol and triethylamine were added to the PVP-coated vaterite particles. This mixture was then cooled to 0 °C to promote the formation of a stable gold coating on the particle surfaces, as depicted in Fig. 2. The surface coverage can be controlled with various linking layers and synthesis temperatures, as we comprehensively analyzed in our forthcoming publication. For scanning electron microscopy (SEM) analysis, the particles were first dispersed in ethanol, and then 5µL of the suspension was deposited onto indium tin oxide (ITO) glass. SEM images of the synthesized particles were captured using a Quanta 200 FEG Environmental Scanning Electron Microscope (ESEM).

A detailed examination of the SEM images in Fig. 2 reveals the successful modification of the vaterite surface before and after the gold nanoparticle synthesis. This uniform size distribution is crucial in ensuring consistent behavior and reproducible optical responses. The particle size distribution was analyzed using the Fiji software^21^, which provides quantitative information about the dimensions of the gold nanoparticles formed under different synthesis conditions. To quantify the coverage of the gold nanoparticles on the vaterite surface, we analyzed the SEM images by calculating the percentage of the surface area occupied by the gold nanoparticles in comparison to the total surface area of the vaterite particles. In Fig. 2a, the surface coverage is ~ 10%, while in Fig. 2b, the coverage reaches ~ 30%. The average size of the gold nanoparticles in Fig. 2a is 9.5 nm ± 6 nm, and in Fig. 2b, it is 12 nm ± 10 nm. The developed approach allows controlling the surface coverage, which can be tuned in a range from 10–30% for the samples synthesized at 0 °C with PVP as a linking layer. Using PVP, PSS, or aminated molecules like APTMS as linking layers and adjusting the synthesis temperature allow for the controlled modification of the vaterite surface. Lower temperatures result in smaller and more uniform nanoparticles, while room-temperature synthesis using a combination of surfactants leads to higher coverage with larger particles. Those were found harder to trap and, consequently, high coverages will not be discussed here. Figure 2c, d demonstrates the particles before and after the surface modification, underlining the change in surface morphology owing to chemical post-processing. Surface roughness was found to have a negligible impact on optical properties. Finally, to ensure that the particles indeed have a gold shell, EDS (Energy Dispersive Spectroscopy) method was used to analyze the elemental composition of materials. Figure 2e demonstrates the EDS data for a particle, verifying the significant gold coverage of its surface. Hereinafter, we will concentrate on the specific 30% surface coverage conditions, as they were empirically found to provide the most stable bubble formation.

As an outlook, the surface functionalization and decoration of vaterite with gold nanoparticles offer significant potential to tune the optical and thermo-optical properties of drug delivery cargoes. Furthermore, owing to facile carboxyl binding schemes, mastered on many occasions, gold can mediate further functionalization of the particle with different functional groups, e.g.^22^.

### Optical properties of gilded vaterite

The infrared (IR) part of the spectrum is preferable for performing experimental studies in a fluid environment owing to the transparency windows of water and, partially, ethanol^23^. The latter solvent will be used in the bubble experiment hereinafter to factor out a probable dissolution of particles during the experiment. Convex small aspect ratio gold nanoparticles have pronounced resonances in the visible part of the spectrum and are less responsive at IR, e.g.^24,25^. Concave shapes, including core shells, are known to provide IR resonances, e.g.^26^. Optical properties of structures encompassing alternating spherically symmetric layers can be addressed analytically with the aid of Mie theory, which allows calculating scattering and absorption cross-sections^27^. In this case, the radii and refractive indexes of shells are the required parameters.

SEM images in Fig. 2 demonstrate a relatively uniform coverage of the vaterite surface with spherical gold nanoseeds. This large number of small resonant particles makes direct electromagnetic analysis to be a very computationally heavy task and thus appeals to approximating models. The concept of metamaterials^28^ comes to the rescue by offering to attribute effective parameters to an approximately homogeneous layer of subwavelength resonators (gold seeds, in our case). While numerous homogenization approaches do exist, e.g.^29–33^, and might consider interfaces, anisotropy, and several other aspects, which are relevant to mesoporous vaterite, hereinafter we will follow a purely phenomenological approach to fit experimental data with a core–shell Mie model, where the thickness of the gold layer is taken as a tuning parameter.

**Figure 3:**
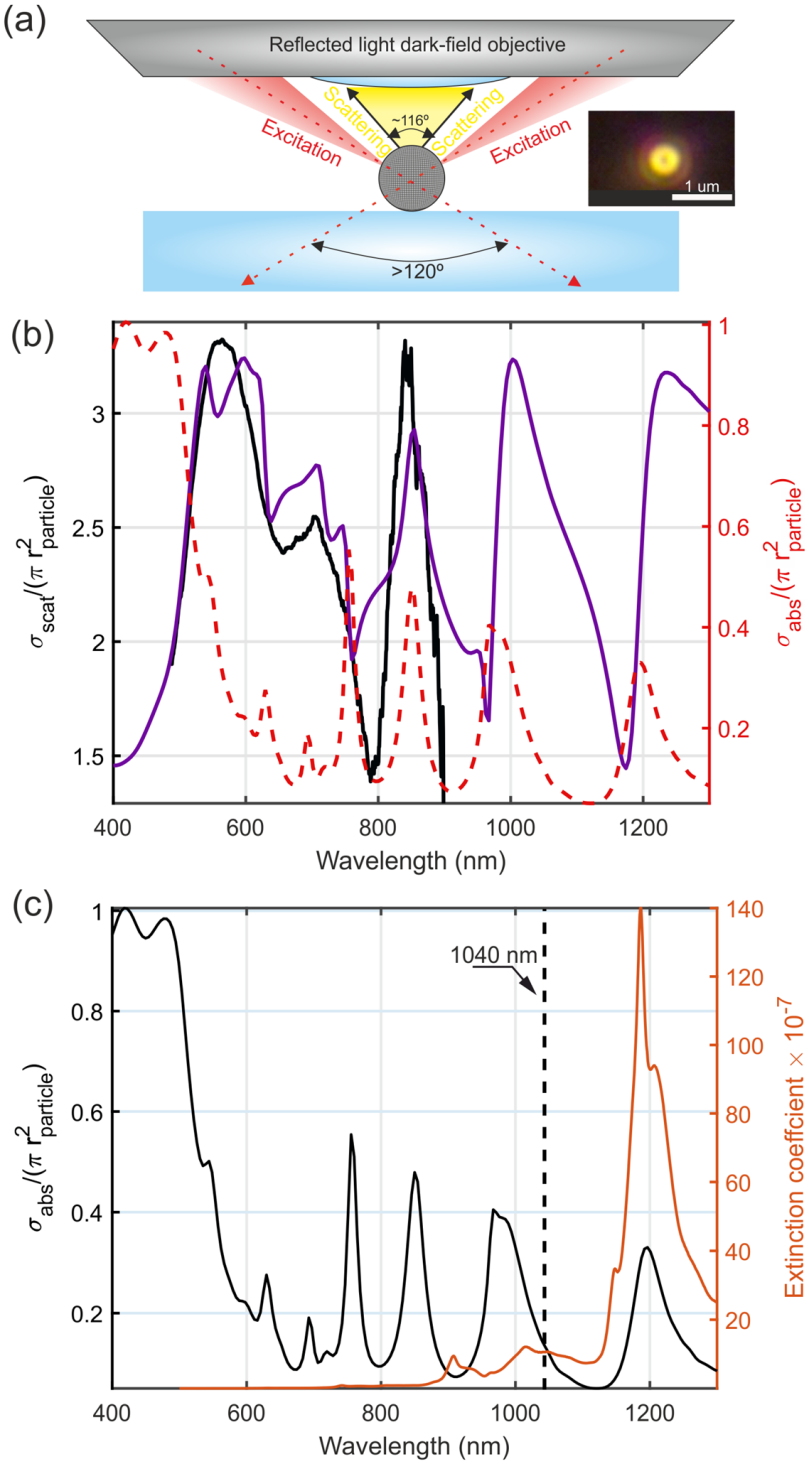
(**a**) Schematics of the dark-field collection optics, including relevant angles. Inset—microscope image of gilded vaterite. (**b**) Optical properties of gilded vaterite in air. Black line—experimental scattering cross-section spectrum, purple line—numerical fit, adapting gold core–shell model (core radius—400 nm, shell thickness—20 nm). The left y-axis (cross-section, normalized to the geometrical size of the particle) corresponds to the data. The experiment is normalized to fit the prediction. The red dashed line with the y-axis to the right is the normalized absorption cross-section. (**c**) Black solid line—the normalized numerically calculated absorption cross-section of gilded vaterite in ethanol (left y-axis). Red solid line—experimentally measured extinction coefficient (imaginary part of the refractive index) of ethanol. The vertical black dashed line at 1040 nm is the central wavelength of a femtosecond source, used for bubble formation.

To investigate the optical properties of the particles, dark-field spectroscopy has been applied^20^. This technique allows for removing the background illumination, solely concentrating on the properties of the scattered field. Our setup uses Fourier plane filtering to separate the illumination and scattering in the k-space of the objective (Fig. 3a). The system was implemented on a Zeiss Axio Lab A1 microscope equipped with reflected light dark-field objective (100x, NA ~ 0.85) to collect scattered light. A representative color image of a particle appears in the inset of Fig. 3a, demonstrating high visibility, thus a sufficient signal-to-noise ratio in the collected spectrum. White calibrated etalon was used to collect the reference signal, which is subsequently used for normalization.

The black curve in Fig. 3b demonstrates the experimentally obtained scattering spectrum. Owing to the collection system arrangement, Ziess microscope lamp and optics allow assessing 550–850 nm spectral range, while longer wavelengths are blocked. The experimental data are normalized to match the subsequent theoretical prediction. To fit the spectral shape, the following parameters of the core–shell geometry have been found empirically—(1) gold shell-20 nm thickness, (2) vaterite particle-averaged isotropic refractive index of 1.6, and the radius 400 nm. The embedding medium was taken as air. In the experiment, the particle is placed on a glass microscope slide [BN1052431STC 24 × 50 mm, thickness 0.13–0.17 mm], which is neglected in the modeling. The purple curve in Fig. 3b demonstrates the numerically calculated total scattering cross-section, normalized to the particle’s geometrical area (πr^2^)—the vertical axis, corresponding to this data, appears on the left. It can be seen that the main spectral features in the experiment are reproduced with the core–shell model. Building on this result, the absorption cross-section can be estimated. It is worth noting that measuring this quantity directly in the case of a single nanoparticle is an extremely challenging task. The red dashed curve on the figure demonstrates the absorption cross-section, where the vertical axis on the right corresponds to the values. This analysis demonstrates that a reliable optical model of the core–shell golden surface vaterite can be constructed. Since the forthcoming optomechanical experiments will be done in ethanol, this embedding medium changes the spectra. Figure 3c demonstrates the absorption cross-section of the particle in ethanol and ethanol’s extinction. The dashed black vertical line at 1040 nm is the central wavelength of the laser, which will be used for trapping and bubble creation. This analysis predicts a close to resonant absorption of the engineered particle, while the heating of ethanol, though it is present, is not the key contributing factor. This statement will be further justified experimentally with a reference (pure) vaterite, which does not support bubble formation at laser powers used in the investigations.

### Bubble formation

Having significant absorption cross-sections, plasmonic nanoparticles are subject to efficient laser heating^34^. Based on this effect, quite a few applications have been assessed, including control of temperature landscapes on a nanoscale^35^, photothermal therapy^36–38^, drug delivery^39,40^, and control over phase transitions^41^, to name a few. Bubble formation around plasmonic nanoparticles, illuminated with laser beams, is under intensive investigation, motivated by bio-related applications, as it can lead to the local destruction of biological tissues or membranes, trigger a shockwave for photoacoustic imaging^42^, and inspire other functions. Femtosecond laser, interacting with a particle in a fluid, might lead to a short-lived nucleation of a bubble^43,44^. Depending on conditions, bubbles can emerge also under continuous wave illumination^45^. The exact mechanism of the formation is still under debate. Several of our observations (e.g., long-living bubbles, which do not collapse after the laser source is switched off) correlate with the seminal report of Baffou et al., where the evidence of overheating and long-living stable micro-bubbles supported the claim that the physical mechanism underlying the interaction is not steam but rather the release of gas molecules into the overheated area^46^. Hereinafter, we will primarily concentrate on the thermal manipulation of bubbles.

**Figure 4:**
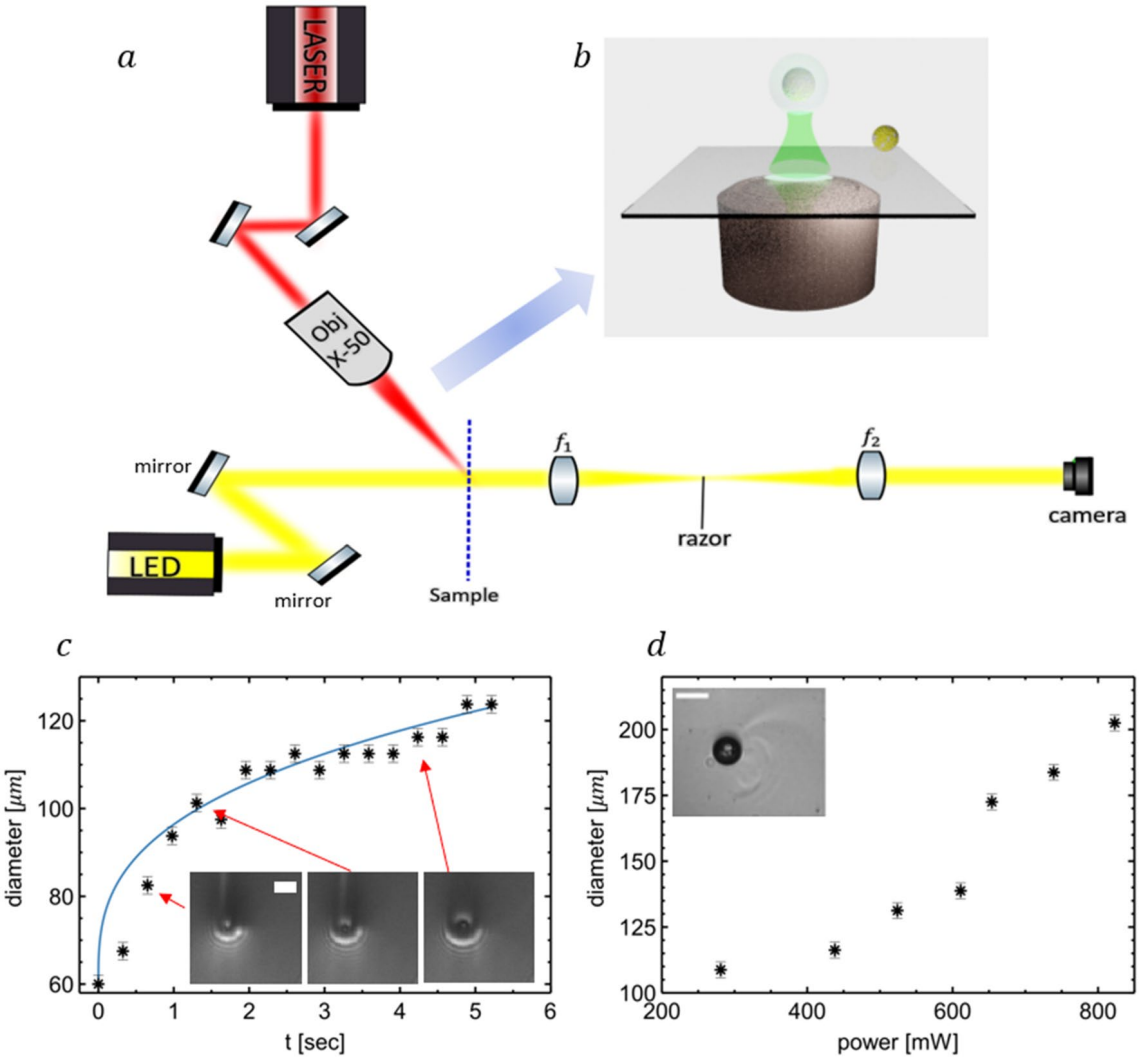
(**a**) 4f Schlieren imaging experimental scheme + femtosecond laser for heating and manipulation of particles in solution. (**b**) 3D schematics of optomechanical manipulation of gilded vaterite in a bubble. (**c**) The bubble size as a function of time. The laser power is kept constant at 438mW averaged power. Black dots—experimental data, blue solid line—theoretical fit. Insets—microscope images of bubbles, corresponding to several points on the plot. White length bar—140 µm. (**d**) Steady-state bubble size as the function of the averaged laser power. Inset—representative microscope image, demonstrating the bubble and the non-isothermal flow around it. The bright dot in the bubble is the gilded vaterite. White length bar—200 µm.

Since the gilded vaterite has a resonant absorption at the IR, extensive heating next to the particle is expected. To trace the non-isothermal flow around the particle, a Schlieren imaging has been constructed^47,48^. The experimental setup (Fig. 4a) is based on a 4f system with lenses of 100 mm and 150 mm focal lengths to compromise between the resolution and the field of view. A razor filter is incorporated in the Fourier space of the 4f system to highlight variations in refractive indices within a sample and visualize the non-isothermal flow. Sharp edges of images allow quantifying sizes of the bubbles and study their formation and time-evolution as a function of laser power, for example. The position of the bubble can also be efficiently tracked with time. Schlieren images were captured with a Flir Grasshopper camera (163 fps, 2.3 MP resolution). Halogen lamp (Avantes, AvaLight-HAL-S-Mini) was collimated on the sample. On top of the 4f system, a MenloSystems YLMO-2W femtosecond laser ($\lambda =1040 \mathrm{nm}$, 120 femtosecond pulses, 2W average power), was introduced. The laser was focused onto the sample, using a Mitutoyo M-Plan X50 objective at a 60° angle of incidence to suppress the scattering into the imaging objective. This laser source is responsible for the heating and optomechanical manipulation of nanoparticles. The laser beam with an initial 2 mm diameter and an intensity of $3.37\cdot {10}^{5}\frac{W}{{m}^{2}}$ is focused by the objective to $7.82\cdot {10}^{9}\frac{W}{{m}^{2}}$, as it was assessed with a power meter (Thorlabs, PM100D) and a calculation. Further control (attenuation) of the beam is made possible with polarization optics (polarization beam splitter and a half-wave plate). Prior to introducing the gilded particle, reference measurements with pure ethanol and uncovered vaterite were performed. No bubble formation has been observed. Numerical estimates suggest that pure ethanol can be heated by no more than 13 °C if no particle is present.

To study the bubble formation dynamics, gilded vaterite particles were mixed with ethanol, drop cast on a microscope slide, and enclosed with another slide, thus creating a fluid cell (2 glasses were glued with epoxy to prevent the ethanol evaporation). Being a nonpolar liquid, ethanol does not dissolve vaterite, thus allowing to factor out probable changes of the particle (dissolution) during the experiment.

The interaction scenario is depicted in Fig. 4b, where the bubble, created around the particle is the subject of optothermal manipulation. At first, we will estimate local temperatures in the vicinity of the particle to verify the conditions that an overheating and the subsequent micro-bubble formation is possible. The starting point is the heat equation^49^:

1 $${\rho }_{L}\left(r\right){C}_{p}\left(r\right)\frac{\partial T\left(r,t\right)}{\partial t}=\nabla \cdot \left(k\left(r\right)\nabla T\left(r,t\right)\right)+Q\left(r,t\right),$$

where ${\rho }_{L}\left(r\right),{C}_{p}\left(r\right)$, and $k\left(r\right)$ are mass density, specific heat capacity, and thermal conductivity. $T(r,t)$ is the temperature and $Q\left(r,t\right)={\sigma }_{abs}I\left(r,t\right)$ is the heat source, corresponding to light absorption. For an estimate, we assume the particle to be uniformly heated by an effective CW laser with an averaged power of the femtosecond source. Ethanol parameters are ($\rho_{L} = 800\frac{kg}{{m^{3} }},C_{p} = 2460\frac{J}{kg \,k}, k = 0.16\frac{W}{m \,K}$), $\sigma_{abs} = 5.2 \times 10^{ - 14} m^{2}$ (estimate from Fig. 2), and $I = 7.82 \times 10^{9} \frac{W}{{m^{2} }}$, the steady solution is $T\left(r>R_p\right)= \frac{{\sigma }_{abs}I}{4\pi kr}+{T}_{0}$, where ${R}_{p}$ is the particle’s radius and ${T}_{0}$ is an ambient (room) temperature. Substituting the numbers, 100 s of °C temperature around the particle is estimated (~ 300 °C at 1 µm distance) thus leading to a bubble formation.

Baffou’s model^46^ distinguishes between 2 types of bubbles: those that are stable for more than 5 s and others that shrink after $\sim$ 100 nsec. Since every liquid contains gas molecules inside unless degassed, it was noticed that bubbles that formed in a very short time tends to shrink, while those that were generated in a longer process are much more stable. Long-living bubbles originate from gas molecules’ diffusion towards the nucleation center. When the femtosecond laser is switched off, it takes up to a few hours for the bubble to collapse. On the other hand, faster-generated bubbles, created by a CW source, contain a vaporized liquid inside, and the structures tend to shrink immediately after the laser is off.

In our experiment, the CW laser was observed to generate short-living bubbles, which shrink right after the beam is removed from the area, as in video 1. For the pulsed laser, bubbles grow slower, and they do not shrink for at least 3 min after switching off the excitation, as in video 2–3. This observation correlates with the report in^50^. It is worth noting that the conditions in^46^ are significantly different from those we have here. Particles and solvents (water vs. ethanol) are very dissimilar and thus may lead to variations in observed effects.

The bubble growth can be described by the Laplace pressure equation:

2 $${\Delta P=P}_{b}-{P}_{l}=\frac{2\sigma }{{R}_{b}},$$

where ${P}_{b},{P}_{l}$ are pressures inside the bubble and liquid, respectively, while $\sigma =0.02\,\frac{N}{m}$ is the surface tension and ${R}_{b}$ is the bubble’s radius. Liquid pressure is the partial pressure, which is exerted by gas molecules inside the liquid. When gas molecules drift into the bubbles, they simultaneously increase the pressure inside, while the liquid pressure drops. As $\Delta P$ decrease, the bubble increase is slower, as shown in Fig. 4c. In this experiment, the bubbles reach a steady state size after ~ 5 s. The size dependence, as a function of the laser power, is represented in Fig. 4d. It can be seen that the bubble size grows with the power increase.

To retrieve the growth dynamic till the steady-state size, a differential equation from^46^ can be used:

3 $$\dot{R_b}=\frac{2DRT\sigma }{K{R_b }^{2}\left({p}_{\infty }+\frac{4}{3}\frac{\sigma }{R_b}\right)} ,$$

where $R=8.314\frac{J}{\text{mol K}}, T=350 K, D=6.76\times {10}^{-9}\frac{{m}^{2}}{s},\sigma =0.02\frac{J}{{m}^{2}}$ are air constant, temperature, molecular diffusivity, and surface tension, respectively^51^. ${P}_{\infty }=101 \,\text{kPa} , K=2100\times 1.83 \frac{\text{Pa m}^{3}}{mol}$ are ethanol pressure out of the bubble^52^, and Henry law’s air coefficient^53^, respectively. Henry’s coefficient was taken as the average value between nitrogen and oxygen^54^, using the conversion value of 1.83 between atm to $\frac{{\text{Pa m}}^{3}}{mol}$. For bubbles above 500 nm, the term $\frac{4}{3}\frac{\sigma }{r}$ might be neglected, and the solution of Eq. (3) is:

4 $$R_b\left(t\right)={R_b}_{i}+\sqrt[3]{\frac{6RDT\sigma }{{P}_{\infty }K}t},$$

where ${R_b}_{i}$ = 30 μm is the initial size of the bubble. The third root of the time law $(\sqrt[3]{t})$ resembles the mechanism of bubble growth, initiated by gas molecules. As the bubble grows, it keeps attracting gas molecules until the depletion thus reaching the steady state^46^ (in this case, assumptions of Eq. (3) do not hold anymore).

The bubble formation was observed in the experiment. Figure 4c demonstrates the evolution of the bubble size (measured from microscope images) for a given laser power, which was kept constant (438mW averaged power). In this experiment, the bubbles growth law followed the Eq. (4) relation with time, before approaching the steady state. The size dependence, as a function of the laser power, is represented in Fig. 4d. It can be seen that the bubble size grows with the power increase.

It is noteworthy that despite the presence of high temperatures capable of melting gold nanoparticles, the gold cover surrounding the particle remained intact in proximity to vaterite. This allowed for the regeneration of a bubble around the same particle more than once, as illustrated in the supplementary information. Interestingly, the growth of the second bubble was notably rapid, presumably due to the pre-existing high temperature of the surrounding liquid. Furthermore, the laser powers required for gold particles damage are orders of magnitude higher to those, which were used in our experiments^55,56^.

### Bubble thermo-optomechanical transport

After demonstrating the bubble formation, the next step is to control its motion with the aid of the optical beam. After switching off the femtosecond laser, the bubble does not collapse and drifts owing to non-isothermal flow till it researches a quasi-equilibrium, undergoing a negligible Brownian motion at an area where no temperature gradient exists. However, once the laser is switched on again, illuminating ethanol (the bubble drifted away), the bubble is consistently pulled back toward the laser focus. This effect is observed even when the bubble is at a rather far 400 µm distance. Obviously, the optomechanical attraction over such a long distance is negligible. Furthermore, the relative polarizability of a bubble is negative, thus it experiences anti-trapping optical forces. The attraction phenomenon here is inspired by the thermocapillary (Marangoni) effect^57–63^. When a liquid is heated, its surface tension decreases, which causes the bubble to be attracted toward the hot spot as long as there is a temperature gradient present in the fluid. The heat distribution inside ethanol, illuminated by a laser beam, has an exponential profile^59^:

5 $$T\left(r\right)={T}_{0}+\Delta \mathrm{T}\cdot {\mathrm{e}}^{-\mathrm{r}/{\mathrm{r}}_{\mathrm{D}} },$$

where ${T}_{0}$ = 23 °C is ambient temperature, $\Delta \mathrm{T}$ is the peak temperature increase, $r$ is the distance between the laser spot to the bubble, and ${r}_{D}$ = 70 μm given the fluid parameters. The thermocapillary effect can be described with the following equation:

6 $${\rho }_{0}=-2\pi {R}_{b}^{2}\nabla T\frac{d\sigma }{dT},$$

where $\sigma =0.02\frac{N}{m}$ is the surface tension of ethanol, $\frac{d\sigma }{dT}=-0.0009\frac{N}{m\cdot k}$ is the temperature coefficient of surface tension^64^, ${R}_{b}$ = 55 μm is the bubble radius, and $\nabla T=-\frac{\Delta T}{{r}_{D}}{e}^{-\frac{r}{{r}_{D}}}$ is the gradient of temperature around the bubble (Eq. 5). Equation 6 states that as long as there is a temperature gradient along the bubble's surface, there will be a thermocapillary effect pulling the bubble along the temperature gradient. The overall force will be around 10nN when the laser is focused 400 μm away from the bubble and it will reach hundreds of nN at 100 μm. Those forces are large compared with pN-scale optical forces and thus prevail over them. Furthermore, buoyancy forces do affect the bubble, but are rather small compared to thermocapillary interactions. Only when the laser was turned off, buoyancy force affected bubble motion, as is video 3.

**Figure 5:**
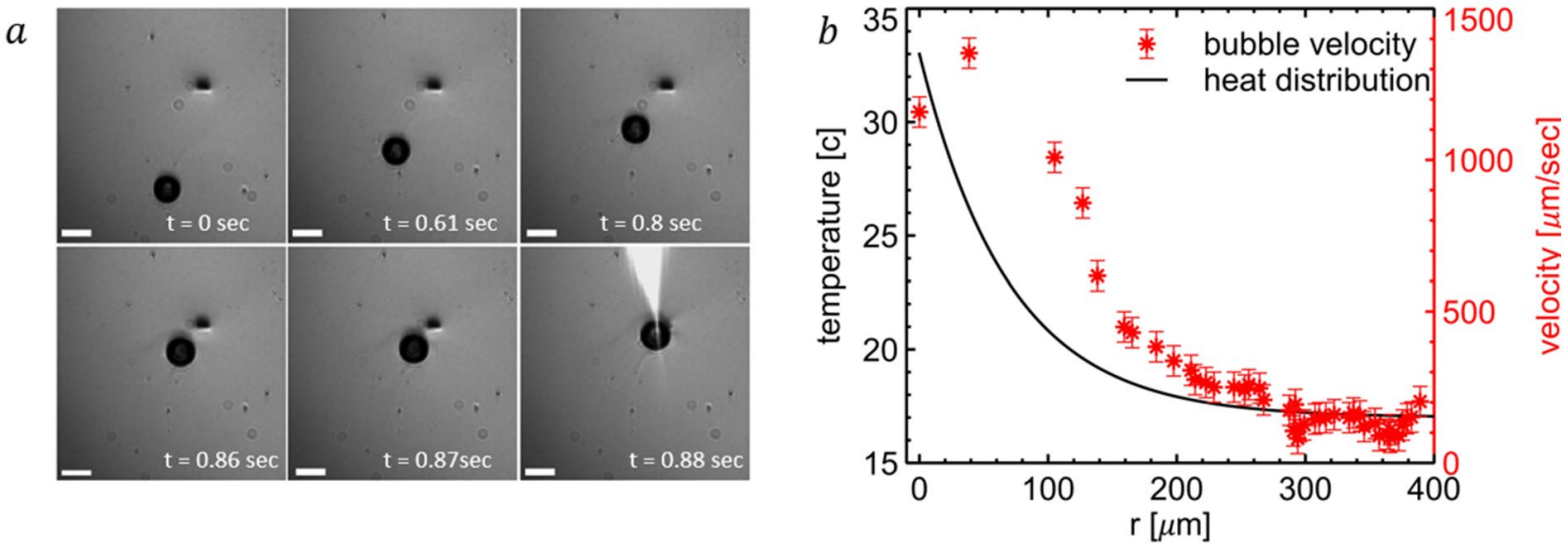
Optothermal attraction of a bubble. (**a**) Schlieren images of the bubble pulled toward laser focus—the black dot. White length bar—180 µm. (**b**) Black line, left y-axis—temperature profile as the function of the distance to the laser focus (analytical model). Red dots, right y-axis—the bubble’s velocity as the function of the distance to the laser focus (experimental data).

Figure 5a demonstrates the Schlieren images of the interaction, where the small black spot is the laser focus. Consecutive timeframes from video 5 are presented, demonstrating the bubble attraction to the laser spot. The bubble’s velocity can then be retrieved, and the results appear in Fig. 5b, demonstrating that the velocity gets larger as the temperature gradient increases close to the laser beam. The velocity profile deviates from exponential fit as additional forces also affect the motion. Buoyancy force (${F}_{b}={\rho }_{L}gV\sim 4nN$ , where ${\rho }_{L}=800\frac{kg}{{m}^{3}}$ is ethanol density, $g=9.8\frac{m}{{\mathrm{s}}^{2}}$ is gravitational acceleration, and $V$ is bubble volume) is $\sim 7\times {10}^{-13} \,\text{m}^{3}$ is rather negligible. However, the drag force (${F}_{d}=6\pi \mu {R}_{b}\cdot v$, for $\mu =1.071\times {10}^{-3}\,\text{Pa s}$ is ethanol’s dynamic viscosity, and $v$ is bubble velocity) affects the motion as it reaches values of several hundreds of nN. Overall, the velocity profile resembles the temperature exponent-like temperature gradient profile.

## Conclusion

Vaterite nanoparticles pose natural biocompatibility and strong porosity, enabling high payload capacity, thus finding use in drug delivery and tissue engineering applications^65–68^. Depending on conditions, vaterite undergoes either a phase transition or dissolution upon interaction with an environment. While this mechanism is responsible for the drug release from the capsule, it has a drawback of an uncontrolled leak. To partially address this issue and also to demonstrate a new efficient light-driven transport of the cargo, we designed a gilded vaterite nanoparticle and interacted it with an IR laser light. Controlling optical properties with the concentration of gold nanoparticles on the vaterite surface allowed for tuning the resonant response of the complex and shifting it to the biological transparency window, as it was verified with dark-field spectroscopy. Interaction between the gilded vaterite in a fluid and femtosecond IR laser was shown to lead to a stable long-living micron-scale bubble, which remained sustainable over minutes after the light source was switched off. Apart from bubble formation around gilded vaterite studies, long-range optothermal attraction of bubble to the laser focus has been demonstrated. The effect was shown to prevail over optical forces by orders of magnitude and it is attributed to the thermocapillary Marangoni effect, associated with the temperature-dependent surface tension.

As an outlook, the controllable bubble can protect the drug capsule from interaction with fluid, thus preserving its properties for longer times. Furthermore, microbubbles are themselves efficient contrast agents in ultrasound imaging and thus can grant the developed platform an additional function on pathways to the paradigm shift towards theragnostic nanodevices^69,70^.

## Supplementary Information

Supplementary Information 1.

Supplementary Video 1.

Supplementary Video 2.

Supplementary Video 3.

Supplementary Video 4.

Supplementary Video 5.

Supplementary Video 6.

## Data Availability

All data generated or analysed during this study are included in this published article (and its Supplementary Information files).
