# Supplementary material for: Gilded vaterite optothermal transport in a bubble
Source: Sci Rep. 2023 Jul 27;13:12158. doi: 10.1038/s41598-023-39068-8 (PMC10374586; doi:10.1038/s41598-023-39068-8)
Supplement: Supplementary file 1 — Supplementary Information 1. [file 41598_2023_39068_MOESM1_ESM.docx]

**Supplementary information**

**Video 1:** Bubble is generated by a CW laser.

**Video 2:** Bubble is generated by a pulsed laser.

**Video 3:** When laser is off, bubble flows away since buoyancy force. It can be seen that bubble does not shrink neither collapse.

It can be seen that bubble growth was much faster in CW laser, as claimed in the paper. Additionally, the bubble shrinks in CW laser, and did not when pulsed laser was used.

**Video 4:** Shows that the heat induced by laser did not melt the gold cover, so after generating a bubble the particle is able to form a second bubble.

**Video 5:** Shows how laser spot is pulling a bubble from distance.

**Video 6:** Shows bubble trapping and manipulation. As the sample is moved, bubble stays in the same spot of image.
